# Severe Sulfuric Acid Attack on Self-Compacting Concrete with Granulometrically Optimized Blast-Furnace Slag-Comparison of Different Test Methods

**DOI:** 10.3390/ma13061431

**Published:** 2020-03-21

**Authors:** Sara Irico, Laurence De Meyst, Dirk Qvaeschning, Maria Cruz Alonso, Kristina Villar, Nele De Belie

**Affiliations:** 1 Wilhelm Dyckerhoff Institut for Building Material Technology, Dyckerhoff GmbH, Dyckerhoffstraße 7, 65203 Wiesbaden, Germany; dirk.qvaeschning@dyckerhoff.com; 2 Magnel-Vanderpitte Laboratory for Structural Engineering and Building Materials, Ghent University, Tech Lane Ghent Science Park, Campus A, Technologiepark Zwijnaarde 60, B-9052 Ghent, Belgium; laurence.demeyst@ugent.be; 3 Eduardo Torroja Institute for Construction Science-CSIC, Serrano Galvache 4, 28033 Madrid, Spain; mcalonso@ietcc.csic.es (M.C.A.); kvillar@ietcc.csic.es (K.V.)

**Keywords:** acid attack, self-compacting concrete, sulfuric acid, acid resistant concrete, granulometric optimization, blast-furnace slag, smart additions

## Abstract

The corrosion by severe sulfuric acid attack at pH 2 of two self-compacting concrete (SCC) types that are based on ordinary Portland cement (OPC) and granulometrically optimized blast-furnace slag cement was evaluated by three complementary tests that were performed in different research institutes. The use of SCC is a smart and promising solution to improve the performance of concrete in an aggressive environment, especially regarding ready-mixed concrete applications, since good compaction is less dependent on workmanship. The relevance and practical advantages of the different test protocols and the influence of the experimental parameters are discussed. It appears that the frequency of renewing the acid solution during the exposure period is the main parameter that influences the mass loss and the rate of degradation, while the sample geometry and the ratio between the volume of solution and concrete surface area had no clear influence. Nevertheless, there was reasonable agreement between the methods regarding the magnitude of the concrete degradation (resulting in a mass loss of about 2.5 kg/m² in six months time). The use of granulometrically optimized slag cement provided a moderate increase of the concrete resistance against acid attack, and this practice might be recommended in order to increase the durability of structures exposed to sulfuric acid media. The fact that the difference in comparison with SCC-OPC was rather limited shows that the influence of the cement type becomes less relevant in the case of concrete with low w/c ratio and optimized concrete technology.

## 1. Introduction

The evaluation of the durability of concrete infrastructures that are exposed to aggressive environments needs a deep investigation of the degradation mechanisms in severe conditions, such as mechanical fatigue, thermal stresses, freeze/thaw cycles, carbonation, soft-water leaching, and chemical attack. Among chemical attacks reducing the service life, acid attack is particularly detrimental for cementitious material due to its alkaline matrix. During an acid attack, the dissolution of the hardened matrix and, if soluble, the aggregate results in the formation of a visible degraded layer, which affects the life cycle performance and the maintenance costs of the infrastructures. Acid attack can be extremely diverse and it is related to specific applications [1]. The severity of acid attack is mainly classified according to the pH and the acid type.

Acidic media with pH lower than 4.5 are considered very aggressive. Nevertheless, the aggressiveness cannot be determined by the pH alone. Degradation by sulfuric acid is considered to be one of the most severe deterioration mechanisms and usually originates from industrial processes, for example: flue gas condensation in cooling towers, geothermal energy generation, mining production processes, wastewater treatment in sewage plants, and transport in sewer pipes.

In the case of sulfuric acid attack, the additional and/or accelerated deterioration occurs because the calcium sulfate formed will affect concrete by a sulfate attack mechanism [2,3], while, in addition, hydrogen ions exert a dissolution effect. The sulfuric acid reacts first with the calcium hydroxide (CH) to precipitate gypsum (CaSO_4_ 2H_2_O) on and below the concrete surface, which can induce tensile stresses in concrete, resulting in cracking and spalling [4]. Nevertheless, if not removed from the surface the accumulation of gypsum can lead to a surface self-sealing of the matrix [5], thus reducing the corrosion rate, especially at early stages. Furthermore, it has been demonstrated that the gypsum precipitation is even more pronounced at pH values that are lower than 2 [6], which implies that a stronger sulfuric acid solution (with lower pH) could produce a smaller weight loss in a concrete specimen than that produced by a weaker solution. Still, at later ages, gypsum precipitation can lead to cracking due to a significant increase in the volume [7]. Furthermore, the reaction with calcium aluminate (C_3_A) will lead to the formation of ettringite (3CaO-Al_2_O_3_-3CaSO_4_-32H_2_O), which has even larger volume and is able to induce micro- and macro-cracks [3]. Once portlandite is depleted, the pH of the pore solution decreases and, along with the dissolution of the AFm and AFt, decalcification of the calcium silicate hydrate phases (C–S–H) proceeds, causing the increase of porosity and degradation of the hydrated matrix. The pH range in which ettringite is stable has been reported to be between 9 and 13.4. It is generally agreed that the stability of the AFm phases increases with the pH of the system [8,9].

**Figure 1:**
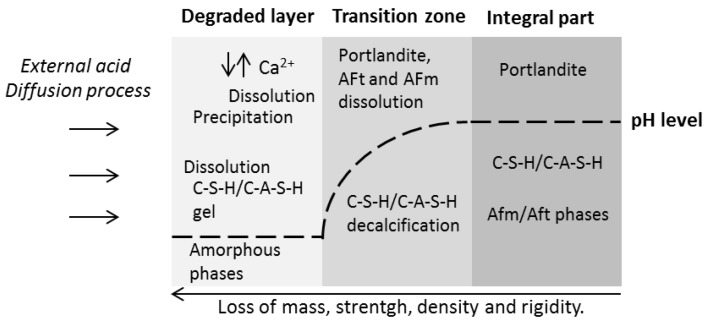
Degradation process of concrete subjected to acid attack showing pH distribution over depth.

In terms of the pH distribution over depth, the degradation process can be described by three zones (degraded zone, transition zone, and intact matrix), which gradually move into the undamaged material as time progresses (Figure 1).

Acid corrosion cannot be measured by only a single parameter, due to the complexity of the phenomenon. The loss of mass, the loss of strength, and change of size at a certain point in time are parameters that are related to the chemical kinetics, which define the degradation rate [10]. Several factors influence the degradation rate of acid attack [11]:

1. Factors that are related to the chemical composition of the matrix (depending on the cement type and use of supplementary cementitious material (SCM), which reduces the Ca/Si ratio within hydrated phases, cement content, mineralogy, thermodynamic stability, stability, and thickness of the corrosion layer;
2. Factors that are related to concrete technology, such as water/cement ratio, curing time, permeability, capillary suction, matrix density, air entrainment, and the influence of the aggregates.
3. Factors that are related to the real environmental condition or the experimental procedure in the laboratory, such as the acid concentration, diffusivity of the acid, mobility of the acidic solution, frequency of acid removal if the pH is not maintained by the addition of acid, mechanical abrasion (brushing or not brushing of the surface in laboratory tests), and the type of indicator for evaluating the acid resistance of the concrete (visual inspection, loss of mass, loss of strength, expansion, and E-modulus).

Strength and impermeability are the essential features to which concrete technology is directed. A high level of impermeability usually also benefits the chemical resistance. A water/cement ratio in the range between 0.35 and 0.40 with cement content of 320 to 360 kg/m^3^ results in relatively high compressive strength and it achieves a high concrete impermeability due to the resulting low capillary porosity. Self-compacting concrete (SCC) is considered to be a smart solution for improving the performance in aggressive environment, especially regarding ready-mixed concrete applications, since good compaction is less dependent on workmanship. Although there has been growing use of SCC in several concrete applications, the acid resistance of SCC still needs to be investigated in depth.

Additionally, the influence of SCM, which is usually incorporated in SCC, must be considered. As recommendation according the ACI 201.2R-01 certain pozzolanic materials and silica fume in particular, increase the resistance of concrete to acids [2]. Indeed, a significant improvement in acid resistance can be achieved by a careful controlled use of ultrafines, in order to have a very dense cement paste matrix [4,12]. The use of CEM III/B blastfurnace slag cements and fly-ash in the concrete mix design are often recommended for improving the acid resistance and representing the actual best state of art in the construction of cooling towers of coal-fired power stations exposed to a high risk of sulfuric acid attack [13,14,15].

The estimation of acid corrosion at lab scale is strongly influenced by the experimental conditions and the effectiveness of the test method, as described above. There are no standard methods that allow for classifying the resistance of a concrete to a combined acid and sulfate degradation and several methods are present in literature based on different experimental conditions like sample geometry, acid concentration, duration of the test, abrasion, or corrosion index [16,17,18,19,20].

When considering that sulfuric acid attack is one of the most complex acid attacks to simulate at the lab scale, the present paper aimed to compare three different test methods developed and performed by different laboratories for testing the sulfuric acid resistance of SCC that was exposed to pH 2 up to seven months. The extreme low pH has been selected in order to evaluate the acid resistance potential of SCC beyond the threshold of pH 4.5 established for the exposure class XA3 by the European Standard EN 206 [21]. The methods that are compared in this study are quasi static and they differ in the type and frequency of stirring, the frequency of solution renewal, the sample geometry, the brushing process, and in the methodology to maintain the pH of the system as close as possible to pH 2. The acid corrosion characterizations at micro and macro level, as well as the degradation rate, are discussed in relation to the experimental conditions.

## 2. Materials and Methods

**Figure 2:**
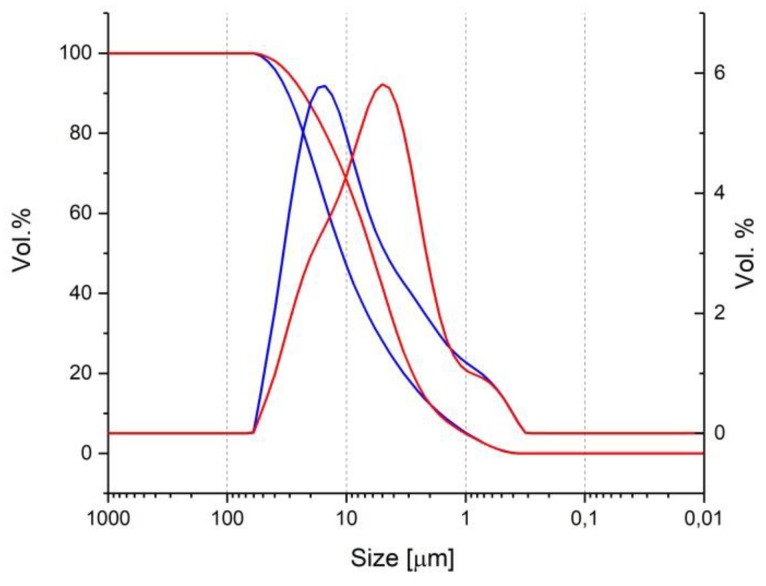
Granulometric curve of the cements: red, SCC-Slag and blue, SCC-ordinary Portland cement (OPC).

Two types of binder were used, including an ordinary Portland cement CEM I 52.5 R and a granulometrically optimized blast-furnace cement CEM II/B-S 52.5 R. Table 1 summarizes the chemical (performed by X-ray florescence) and mineralogical analyses (performed by X-ray diffraction) of the used binders. The granulometric analysis of the binders, as in Figure 2, was performed by laser diffraction.

Two SCC were produced according to the mix design depicted Table 2. In this study, class C fly ash was used as supplementary cementitious material to improve the rheological properties of SCC and the matrix density. The total binder content was kept constant and it conforms to common SCC mixture guidelines [22]. The aggregate type was siliceous to guarantee its resistance to acid environment. The specimens were produced and cast in the same lab and distributed to the other laboratories after a water curing period of 28 days in order to ensure the reproducibility in the concrete preparation.

The compressive strength at 28 days was determined for each mixture on two cubes (150 × 150 × 150 mm^3^). The concrete specimens were cured until seven days in water and 21 days at 20 °C and 65% relative humidity. The static elastic modulus was measured on cylinders (diameter 150 mm, height 300 mm) according to the EN 12390-13 [23]. The capillary water sorption was measured on concrete cubes (150 × 150 × 150 mm^3^) that were stored for seven days at 20 °C and 65% relative humidity and then put in contact with 5 mm water for 21 days. The pore size distribution of the concrete was measured by mercury intrusion porosimetry under vacuum while using a Micrometrics Autopore IV 9500.

### 2.1. Constant pH Method

In this method, identified as “constant pH method”, performed at the Wilhelm Dyckerhoff Institute at Dyckerhoff GmbH, the pH is kept constant at pH = 2.0 ± 0.2 with H_2_SO_4_ during the testing period. The concrete cubes (150 × 150 × 150 mm³) were cut in slices (150 × 150 × 40 mm³) in order to remove the surface that was in contact with the mold.

**Figure 3:**
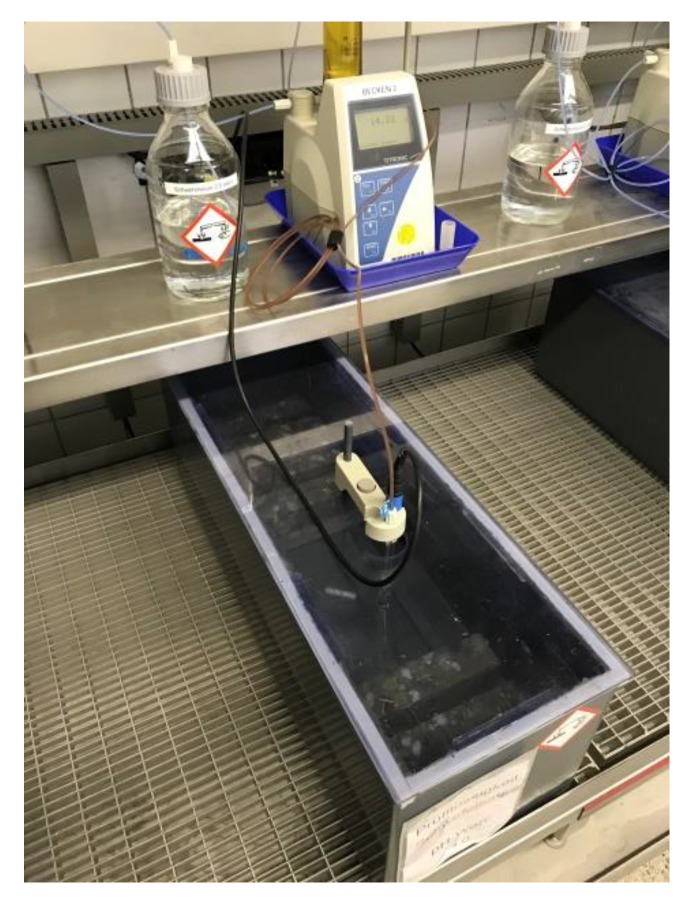
Constant pH method.

The concrete prisms were immersed in 12 L of water; afterwards, the titrator (Schott Titronic® Universal, East Bunker Ct Vernon Hills, IL, USA) pumped the sulfuric acid into the container until pH 2 is reached (amount depending on the alkalinity of the concrete). The pH value in the reservoir was continuously controlled and adjusted via an automatic titrator. A magnetic stirrer (Heidolph, MR3001, Schwabach, Germany) distributed the concentrated sulfuric acid (H_2_SO_4_, 2.0 molar), which is automatically trimmed in the reservoir when required (Figure 3). The test solution in the reservoir and in the storage tank was replaced every week at least until 35 days; afterwards, the solution was renewed every month. The concrete slices were subjected to a manual brushing in water before measuring the acid degradation, as recommended by [17].

Subsequently, they were weighed after drying the surface with an adsorbent paper. The degradation was measured by the loss of mass in kg/m^2^ to allow for comparison samples with different geometry. According to Breit [12], the sample geometry has no relevant influence on the area-related test results of sulfuric acid attack if a constant ratio between liquid volume and sample surface area is maintained.

In this test protocol, the ratio of the acid solution volume, Va, and the sample external geometric surface area, S, was maintained at Va/S = 40 mm during the test. The Impulse Excitation Technique was used as a non-destructive technique (DIN 1048 [24]) for the evaluation of the damage by the decrease of the dynamic E-modulus (N/mm^2^). Scanning electron microscopy (SEM, Leica Cambridge Stereoscan S360, Morgan Hill, CA, USA) was performed for an optical examination of the damage.

### 2.2. Constant Sulfate Method

**Figure 4:**
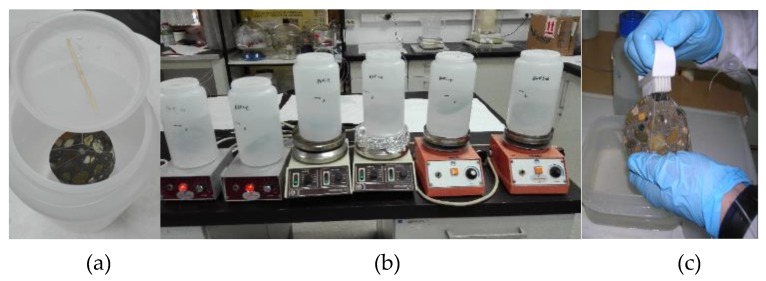
Constant sulfate method-sample immersed in the acid medium (**a**), daily stirring (**b**), and brushing (**c**).

The so-called “constant sulfate method” refers to concrete immersed in an acid solution with initial pH of 2 (Figure 4). The High Research Council of Spain (CSIC) performed the test. Cylindrical cores were obtained, with a diameter of 75 mm from a 150 × 150 × 150 mm³ concrete cube. After eliminating the top and bottom external surfaces (10 mm), slices of 20 mm height were used (final size sample size 75 × 20 mm²). The specimens were vacuum saturated with water according to the method that was described in EN 12390-11 [25]. The specimens were located in a vacuum container and the absolute pressure was reduced to a value between 10 mbar to 50 mbar (1 kPa to 5 kPa). After three hours, the container was filled with distilled or demineralized water by a vacuum pump, so that all of the specimens were immersed. The vacuum was maintained for an hour more before allowing for air to re-enter the container. The specimens were kept in the solution for 18 ± 2 hours.

Six slices of each concrete type were immersed in sulfuric acid that was separated in individual plastic acid-resistant containers. The samples were completely immersed in the acid solution, so that the entire surface of each sample was uniformly exposed to the acid. Two different procedures to remove the degraded external material were performed: a) through manual brushing (four samples), and b) by sonication for 30 s in deionized water (two samples). The volume of acid solution was constant, so that the ratio of the acid solution volume, Va, and the sample external geometric surface area, S, was maintained at Va/S =100 mm during the test. This acid solution was stirred daily during 30 min. and its pH was manually controlled. The acid solution was replaced when necessary to maintain the pH in a range between 2 and 4 (the acidic solution was prepared with 1355 g of deionized water and 0.380 ml of sulfuric acid 95–97% to obtain an aqueous sulfuric acid solution with a Ph = 2 and 426 ppm SO_4_). The concrete damage evolution was followed weekly by the mass loss measurement after brushing or sonicating the samples. The calcium that was released from the concrete to the acid solution was analyzed by inductively coupled plasma optical emission spectrometry (ICP-OES). After three and six months of acid exposure, one specimen of each concrete and cleaning procedure was extracted and then examined using optical microscopy.

### 2.3. Accelerated TAP Method 

**Figure 5:**
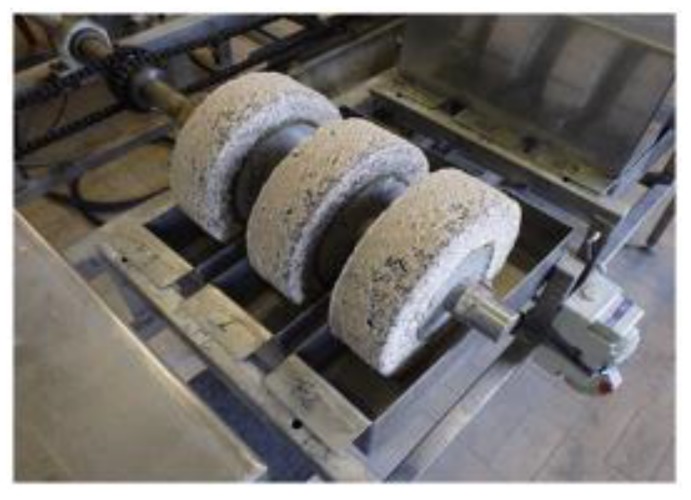
Example of concrete degradation with the accelerated TAP method.

To simulate an accelerated acid degradation, the so-called TAP (Test for Accelerated degradation) experiment, performed at Ghent University, uses alternating wetting and drying cycles since acid penetration by adsorption is faster than by diffusion (Figure 5). This is a realistic and severe situation that can occur, for instance, in gravitational sewer pipes with a fluctuating sewage level. The cyclic wetting and drying is obtained by mounting concrete cylinders on slowly rotating horizontal axles, with the lower part being immersed in individual containers with simulation liquid, such that a point on the circumference would be in contact with the solution during one-third of the time. Next to the wetting and drying, mechanical abrasion by brushing the samples was also included in this test, by three brushes rotating at a speed of 394 r.p.m. that can be mounted on the frame of the TAP. The acid degradation was accelerated to obtain results in a more rapid way by combining both techniques. 

More specifically, the TAP apparatus consists of different rotating axles, with three cylinders of the same concrete mixture on each axis. The test cylinders (Ø 230 mm, h = 70 mm) were cast in dedicated molds containing a central metal tube, which allows for mounting the cylinder on the axles [18]. Two different rotation speeds can be selected: a high speed of 24.41 revolutions per hour and a low speed of 1.04 revolutions per hour. The low speed was chosen during the chemical attack, while the high rotational speed was selected for brushing and measuring. During the chemical attack, the cylindrical specimens were during six days rotating through a recipient with two liters of sulfuric acid solution with an initial pH of 2 (2.67 g sulfuric acid 96% per 2 l tap water). The pH of the sulfuric acid was measured every day (except during the weekends) and replaced every week. Concrete degradation, as measured every week as well, both before brushing and after brushing of the specimen to separate the expansion that is caused by sulfate attack from the volume reduction by mass loss. Degraded material at the surface of the specimen was removed by brushing.

The concrete degradation was measured as the change in radius of the cylinder and by the surface roughness. These measurements were undertaken with laser sensors (ILD 1302-100, VistaLink nv, Mechelen, Belgium) that were mounted through an accurate positioning device on the frame of the TAP and connected to a data acquisition system. By comparing the measured profiles (four per cylinder) that were taken at the start of the test and after each chemical attack cycle of one week, the average change in radius with time was calculated. Additionally, the evolution of the surface roughness with time is an output of this test. The surface roughness is expressed by means of the Ra-value that is based on the British Standard BS 1134, which describes the average departure of the profile above and below the center line throughout the reference length, which was set at 10 mm to exclude the waviness effects from the roughness calculations.

### 2.4. Comparison of the Three Test Methods

Table 3 provides a schematic comparison between the three test methods.

## 3. Results

**Figure 6:**
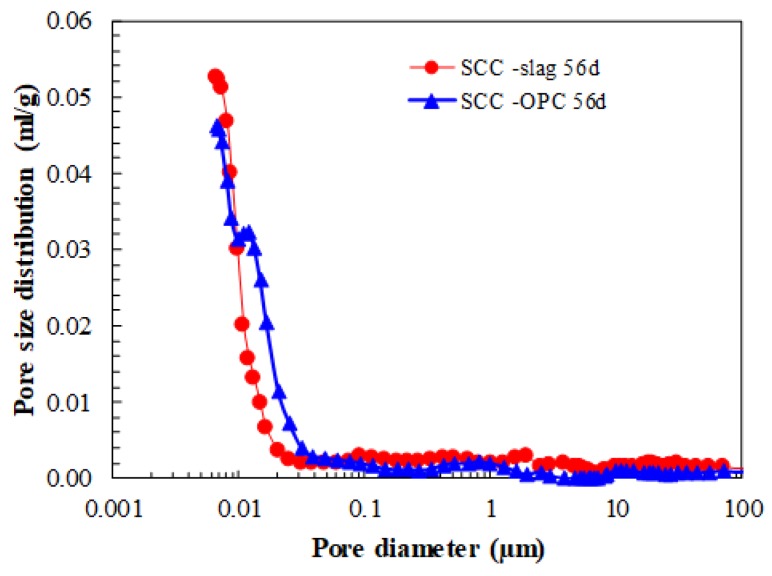
Pore size distribution of the two SCC concrete types.

The SCC produced with granulometrically optimized slag cement (SCC-Slag) has a denser matrix than SCC-OPC, as can be seen in Table 4. This has, as a consequence, an improved compressive strength and E-modulus after 28 days of hydration. As shown in Figure 6, the SCCs have a very low capillary porosity. After 56 days of hydration, the pore size distributions evidence for both concrete types a comparable amount of pores that are smaller than 0.01 µm, which represents the space between the C-S-H sheets. The presence of the granulometrically optimized slag cement reduces the total porosity slightly; in both cases, the matrix is very dense.

Overall, the mechanical and microstructural properties of both concrete types are very similar, although some improvements are obtained with the incorporation of mineral additions in the SCC-Slag concrete.

The characterization of the acid corrosion degradation by means of the different adopted approaches is described in the following paragraphs. 

### 3.1. Constant pH Method

**Figure 7:**
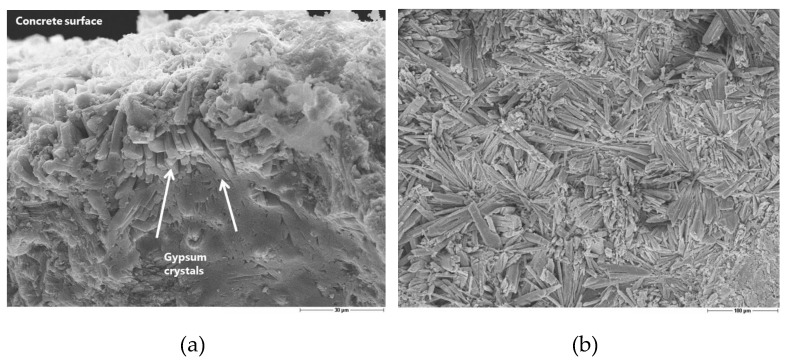
(**a**) Section of a SSC-Slag sample immersed in acid solution for 7 days (scale 30 µm); (**b**) Large gypsum crystals on the surface of the same sample, as shown in (**a**) (scale 100 µm).

In this method, the concrete prims are immersed in sulfuric acid solution at a constant pH of 2. During the first weeks, a high precipitation of gypsum occurs on the concrete surface due to the continuously increasing sulfuric acid concentration that is necessary for maintaining the pH constant and, consequently, increasing sulfate concentration. The prismatic shape crystals of gypsum induce an expansive reaction with a consequent cracking. Anyway, especially during the first week, the precipitated gypsum creates a sort of passivation layer that induces a dormant period (Figure 7). The crystals of gypsum formed deep in the matrix and even bigger crystals are directly observed on the concrete surface, as shown in Figure 7. Indeed, the mass of the concrete increases due to gypsum accumulation during the first week of measurement, even if the surface of the concrete was brushed before weighing the samples. If the concrete surface is not brushed, the gypsum continues to precipitate and grow and the dormant period can be even longer. Depending on the sulfate concentration, following main scenarios can be discerned [21]:

- **high [SO_4_^2-^]** → Ca(OH)_2_ + H_2_SO_4_ **→** CaSO_4_ 2H_2_O (gypsum) → dormant period with precipitation → expansion
- **low [SO_4_^2-^]** → 3CaO Al_2_O_3_ 12H_2_O + 3(CaSO_4_ 2H_2_O) + 14H_2_O **→** delayed ettringite formation → expansion

**Figure 8:**
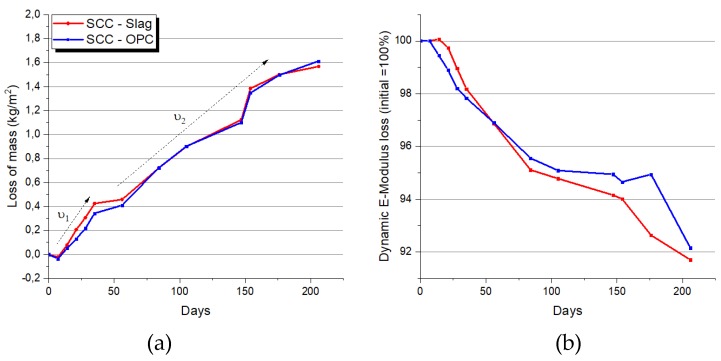
(**a**) Mass loss (kg/m²) during seven months, with respect to initial dry sample weight; (**b**) dynamic E-modulus decrease due to the acid attack.

It was observed that the kinetics of the degradation were strongly dependent on the frequency the solution was changed. During the first months, the solution was renewed every week, whilst afterwards every months, which explains the faster kinetics of loss of mass in the initial period ʋ1, in comparison with the subsequent period ʋ2, which is clearly evidenced in Figure 8. The same kinetics trend was observed for the loss of dynamic E-modulus due to the acid corrosion. 

**Figure 9:**
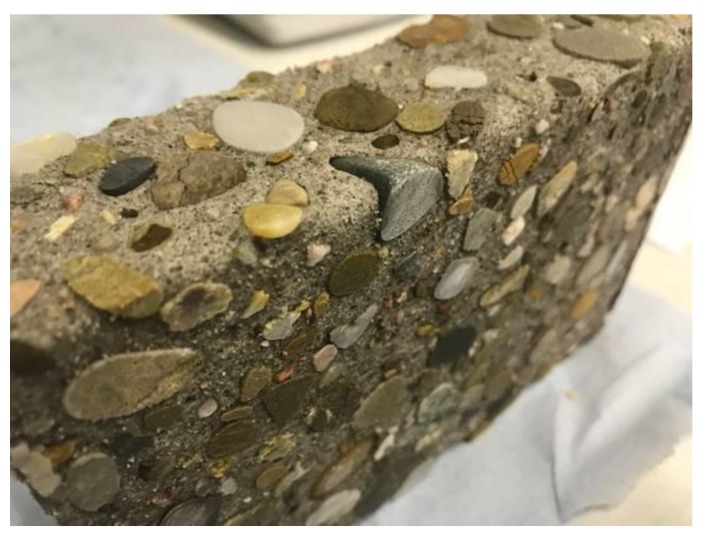
Corroded concrete prism of SCC-Slag after seven months of exposure to sulfuric acid of pH 2.

Except for the slightly lower loss of mass of the concrete SCC-OPC in the initial period, which was probably due to the higher gypsum precipitation of this concrete at early stage, no significant differences are observed between the two concrete types. Figure 9 shows an example of a degraded concrete prism after seven months exposure. The leaching of calcium ions, as measured by the “constant sulfate method” that will be discussed next, showed comparable amount of calcium ions released during the immersion in sulfuric acid. According to these results, the cement type has a minor influence on the SCC resistance against acid corrosion. The high quality of the concrete, with high compressive strength, high matrix density, good consolidation, and the low w/c ratio leads to a negligible effect of the type of cement used.

**Figure 10:**
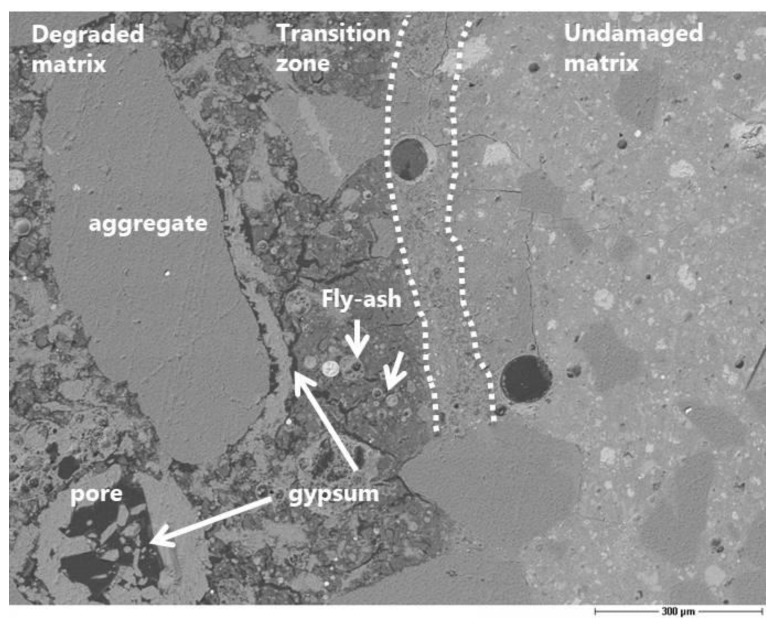
Scanning electron microscopy (SEM-BSE) image of SCC-Slag showing the precipitated gypsum within the interface layer and pores, undamaged fly ash, the transition zone, and the undamaged matrix (scale 300 µm).

**Figure 11:**
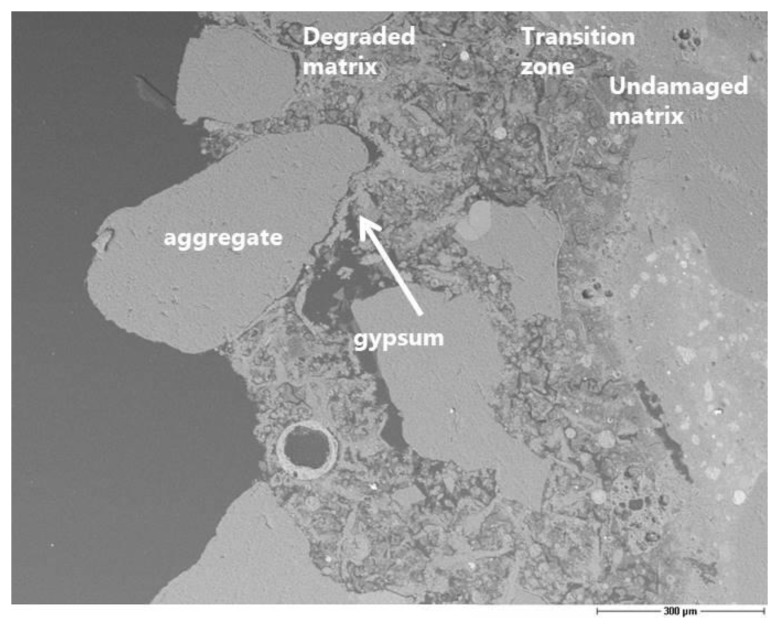
SEM-BSE image of SCC-OPC showing the precipitated gypsum within the interface layer and pores, undamaged fly ash, the transition zone and the undamaged matrix (scale 300 µm).

The evaluation of the corrosion by means of microscopy is useful for understanding the mechanism of sulfuric acid attack, although it is difficult to obtain a quantitative assessment of the damage. The preparation of a thin section is a suitable solution to obtain good scanning electron microscopy images. Nevertheless, it would be necessary to have much more than one thin section per concrete type to guarantee the reproducibility of the corrosion depth measurement along the surface in contact with the acid. Furthermore, the depth of penetration is strongly related to the size of the aggregates. The precipitation of gypsum crystals at the interface between the aggregates and the matrix was clearly observed (Figure 10, Figure 11). This means that the acid diffusion preferentially occurs along the path around the aggregates in the near-surface zone of the concrete. Bassuoni et al. [4] recommend in the case of SCC mixtures an increased volume of coarse aggregates to reduce the mass loss by minimizing the cementitious surface that is available for the reaction with acid. The SEM-BSE (Scanning Electron Microscopy with Back-Scattered Electrons) investigation also confirmed the high stability and resistance of the fly ash to the acid aggression.

### 3.2. Constant Sulfate Method

**Figure 12:**
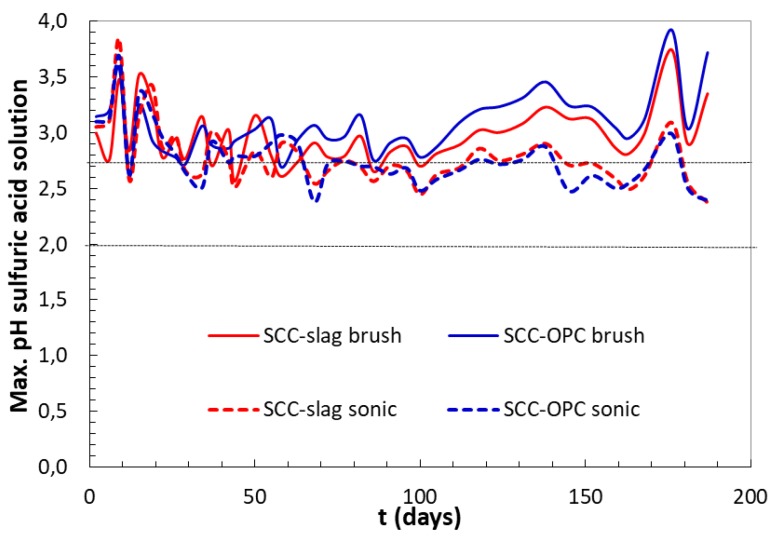
Evolution in time of maximum pH value of the sulfuric acid solution in contact with concrete samples.

The sulfuric acid solution was initially set at pH 2, but, after the contact with the cementitious matrix, the pH increased due to the alkaline character of the concrete, as shown in Figure 12. The increase was faster at the beginning of the test; indeed, a frequent renewing of the acid solution was necessary, daily during the first week, then every 2–3 days, and then weekly. However, the pH of the acid solution was always maintained below 4. During the first 60 days, the pH was quite independent from the type of binder or the surface cleaning method. Afterwards, differences in the measured pH were observed when comparing the brushed samples and the sonicated ones. It can be highlighted that, when compared to the previous method, the concentration of sulfate in the solution was lower, since the solution is weakly renewed and no automatic pH titration is adopted.

The brushing surface cleaning method showed higher pH changes, especially in the case of the Portland cement based SCC-OPC; this could be the consequence of higher portlandite content and, hence, an increased hydroxide leaching. Furthermore, this could induce more gypsum precipitation, due to intensive decalcification of the C-S-H and portlandite, as observed in the “constant pH method”. 

**Figure 13:**
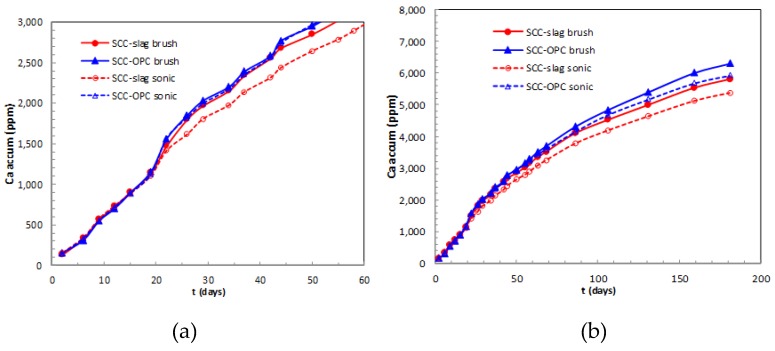
Accumulated value of Ca released from the concretes exposed to acid solution (**a**) during the first 50 days; and, (**b**) until 180 days.

The increased amount of Ca^2+^ ions in the solution confirmed the increase in pH of acid solutions in contact with the OPC after 60 days of exposure due to portlandite dissolution. Figure 13 shows the average values of accumulated amounts of calcium ion released from the concrete samples to the acid solution. A slower pH increase with the sonicated surface cleaning method was measured for both concrete types, indicating lower aggressiveness of the test. In this case, the corroded surface that acted as a sort of passivating layer reduced the contact between the acid and the matrix, and less Ca^2+^ ions were released. The brushing eliminated a larger amount of this reacting layer, exposing new alkaline concrete surface and accelerating the pH increase of the acid solution. 

The released Ca^2+^ present in portlandite, C–S–H gel and AFm/AFt compounds can be an index that represents the corrosion rate. During the first 20 days of acid attack, the Ca^2+^ released is very similar in the two concrete types; no differences are expected from the method whatever the ratio between the acid volume/concrete surface, the concrete size and geometry, or surface cleaning system used. After the first 20 days of testing, the amount of Ca^2+^ ions released from brushed concretes is greater, this explains the increase of pH at longer ages (Figure 12). It was observed with the advance of the test that the dissolution of calcium phases is smaller in the concrete with slag, when compared to SCC-OPC. At early ages the Ca^2+^ is probably depleted from portlandite as first source and then from C–S–H.

**Figure 14:**
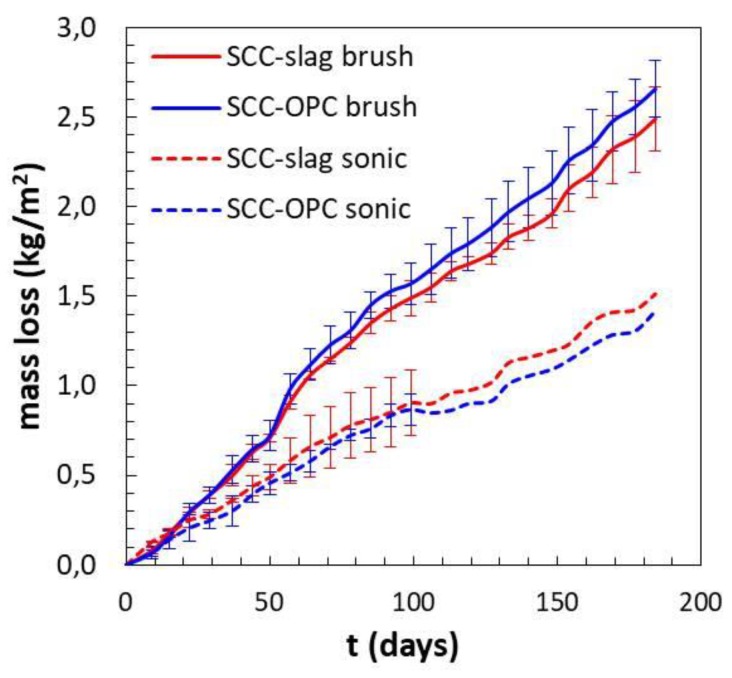
Loss of mass of concrete samples during 6 months of acid attack.

**Figure 15:**
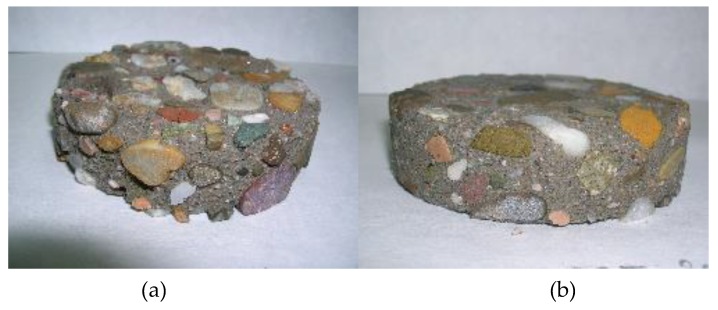
The corroded concrete slices after 3 months in H_2_SO_4_ at pH 2–4: (**a**) SCC-Slag concrete samples brushed; (**b**) sonicated.

The loss of mass of the concrete samples evolves quite linearly with exposure time. The following observations can be summarized:

1. a similar mass loss during the first 20 days was noticed for both concrete types with a minor influence of the cleaning system, which is in agreement with the observed Ca^2+^ release;
2. after two months the influence of the surface treatment started to be relevant, when brushing the surface more material is removed, which is also illustrated in Figure 14 and Figure 15;
3. at later stages, the SCC-OPC had a slightly higher loss of mass compared to the SCC-Slag, this trend and the kinetics is in agreement with the increase of the pH and the release of Ca^2+^ ions in the solution; and,
4. the scatter for each concrete type and surface treatment shows the lower effect of the former in comparison with the latter. After three months, no standard deviation can be shown for the sonication method, as only two samples were employed.

**Figure 16:**
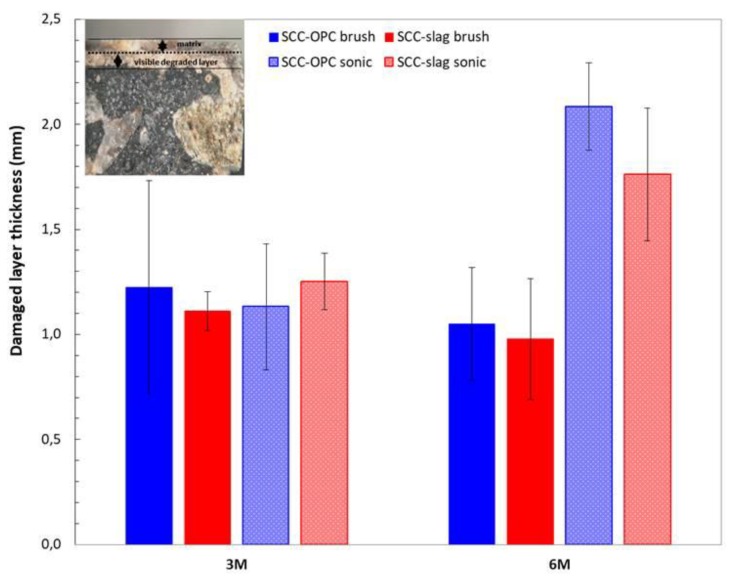
Change in the thickness of the degraded layer of SCC-Slag and SCC-OPC concrete after three and six months of acid exposure.

The samples exposed for three months and six months to the sulfuric acid solution were cut and the fractured surface analyzed with optical microscopy to identify the depth of the corroding layer. The thickness of the degrading layer was measured after three and six months of exposure and it represents the part of the matrix still present that has been attacked by the acid; it is not related to the thickness of the lost layer (Figure 16). Indeed, in the case of the brushed surfaces, the thickness of the degrading layer remained the same, while the loss of mass increased in time. That means that a degraded layer of around 1 mm thickness was always present and moved continuously deeper into the matrix with mass loss as a consequence. Brushing is a more powerful method than sonication and it allows for applying a stronger mechanical action. When the surface was sonicated, the external layer was only partially removed. Indeed after six months of exposure the degraded layer has increased in thickness. The degraded layer of the SCC-OPC was a little thicker than for SCC-OPC due to the higher dissolution of portlandite and C-S-H phases.

### 3.3. TAP Method

In this accelerated acid attack test, two cylinders of SCC-OPC were tested for 12 acid attack cycles, whereas for the SCC-Slag concrete three cylinders were tested during 25 acid attack cycles. The pH of the sulfuric acid solution was set at pH 2 at the beginning and was regularly measured. The solution was replaced every week. The degradation was evaluated by a visual inspection, the change in radius, and the surface roughness of the cylindrical specimens. 

**Figure 17:**
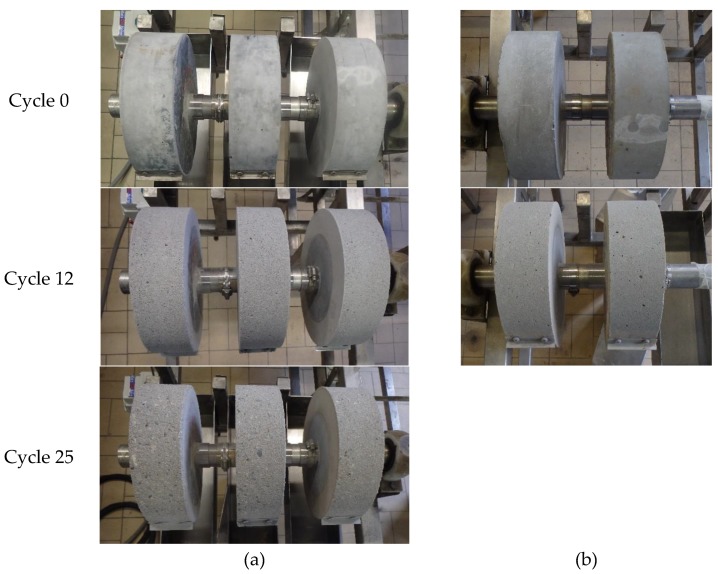
Image of concrete test specimens before start of the test and after different acid attack cycles: (**a**) SCC-Slag; and, (**b**) SCC-OPC.

**Figure 18:**
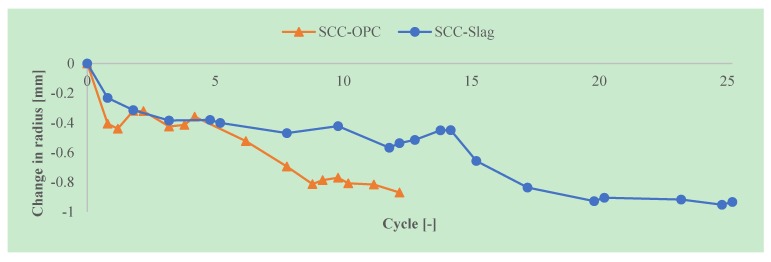
Average reduction in radius of the test specimens exposed to sulfuric acid with initial pH of 2 and weekly solution replacement.

It can be seen in Figure 17 that the specimens were slightly damaged after twelve cycles; however, no visual differences could be observed between the SCC-Slag and the SCC-OPC specimens. After 25 acid attack cycles, the damage of both SCC became more pronounced and the aggregates were clearly visible at the surface. Figure 18 depicts the average accumulated reduction in the radius after every attack cycle of the concrete specimens. Until five acid attack cycles, there was almost no difference between the results from the SCC-Slag and the SCC-OPC specimens. From five acid attack cycles onwards, the SCC-OPC specimens showed a larger accumulated change in radius than the SCC-Slag specimens. It seems that the slag containing specimens showed a higher chemical resistance towards acid attack when compared to the OPC specimens up to 12 cycles. The accumulated reduction in the radius after 12 cycles was around −0.9 mm for the SCC-OPC and −0.5 mm for the SCC-slag concrete. The degradation of the SCC-Slag specimens was around −0.9 mm after 20 acid attack cycles. However, the difference in degradation between the cylinders of the same concrete type was relatively high (e.g., standard deviation on the mean of the three SCC-Slag cylinders being about 0.16), which reduces the significance of the measured differences.

**Figure 19:**
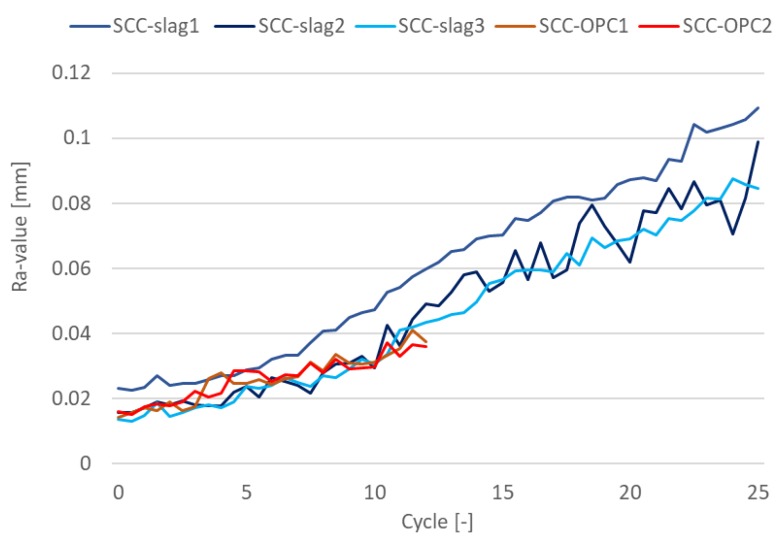
Surface roughness of the test specimens exposed to sulfuric acid with initial pH of 2 and weekly solution replacement.

Besides the degradation depth, the surface roughness was also evaluated, as shown in Figure 19. For both concrete types, the roughness increased at the same rate and almost linearly with time; the change in Ra-value amounted to 0.02 mm during the first 10 cycles. Subsequently, the roughness increased more rapidly with 0.04 mm over the next 10 cycles (only measured for SCC-Slag). Cylinder 1 of the SCC-Slag showed a larger initial surface roughness as compared to the other four cylinders, but the change in Ra-value with time was similar as for the other cylinders. The change in roughness could be determined with good repeatability: for the 12 profiles measured over the three cylinders of SCC-Slag, the standard deviation on the average change in roughness varied from 0.001 mm to 0.004 mm for increasing number of cycles.

**Figure 20:**
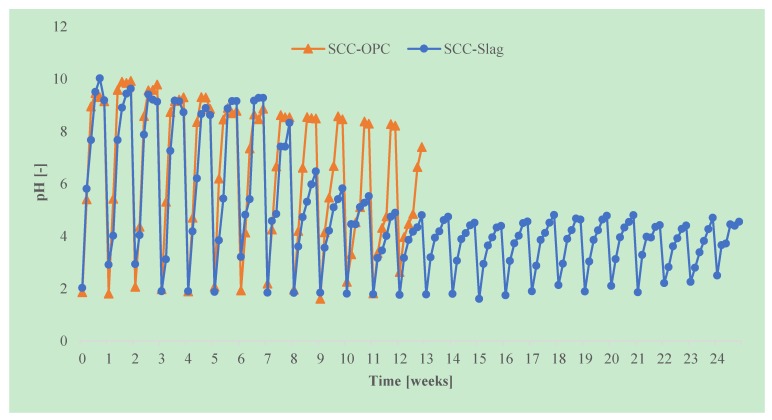
pH of the test liquid during the test (initial pH: 2).

The pH was measured daily and after a week the testing solution was replaced. It was observed that the pH of the solution increased rapidly during a test cycle, as part of the portlandite was leached out of the concrete and neutralized the acid solution, as shown in Figure 20. Even for the slag specimens, which contain less portlandite than OPC specimens, the pH reaches a maximum of pH 10 during the first weeks of the test. However, in each subsequent attack cycle, the maximum pH that is reached decreased, as part of the ions had already been leached out from the concrete surface layer previously and the neutralizing effect was reduced. After eight cycles, the maximum pH of the solution during a cycle decreased to pH 6 for the slag containing specimens, the maximum pH for the OPC mixtures however still rose above pH 8. From cycle 11 onwards, the maximum pH of the solution in case of SCC-Slag decreased to pH 5. The maximum pH of the solution containing the SCC-OPC specimens decreased at a slower rate. When compared to slag containing specimens, OPC concrete has a higher neutralizing capacity, which is due to the higher amount of portlandite present. The rapid leaching of the portlandite leads to faster acid degradation of the SCC-OPC, and this cannot, in this case, be counteracted by the fact that the solution pH is more rapidly reaching a non-aggressive level. This is consistent with the slightly higher radius reduction of the SCC-OPC due to the acid corrosion than that of SCC-Slag.

In previous sulfuric acid degradation tests with the TAP apparatus on samples that were taken from commercially produced sewer pipes [19] a 0.5% sulfuric acid solution was used, leading to an initial pH of 0.9 to 1. Thanks to the much higher acid concentration, the pH remained more constant in this case, and it did not rise with more than 0.7 units during one cycle. Still, after 10 cycles, the decrease in radius of the tested cylinders was not more than 0.4 mm, which is similar to the current results. Some of the tested concrete compositions presented similar characteristics to the currently tested mix designs: gravel aggregates, 350 kg/m³ CEM I 42.5 or CEM III/B 42.5, w/c-ratio = 0.36-0.40, a 28 days compressive strength of 85 N/mm², and water absorption of 3.6%. However, in this case, high sulfate-resistant cement types with lower C_3_A content were used, so this might have compensated for the more aggressive acidic conditions. The difference in degradation between samples with CEM I or CEM III/B was also limited [19], whereas the aggregate type (gravel versus limestone) and concrete porosity (related to the w/c-ratio) had a pronounced effect. Therefore, a model was proposed for predicting the concrete degradation based on the concrete alkalinity (calculated from the CaO content) and the water absorption, specifically for the case of biogenic sulfuric acid corrosion. On the other hand, it should be mentioned that in another TAP investigation by the same authors on concrete produced in the laboratory, the specimens with CEM III/B 42.5 HSR/LA showed clearly improved durability in comparison with the specimens with CEM I 42.5 HSR/LA [20]. The authors ascribed the difference between the two sets of experiments to the more limited curing for commercial precast concrete pipes in comparison with the laboratory specimens that underwent 28 days of ideal curing. It is well known that short curing times more negatively affect the properties of concrete with blast-furnace slag cement than for concrete with Portland cement.

## 4. Discussion

The evaluation of the concrete corrosion by the exposure to sulfuric acid is much more complex when compared to other types of acid due to the combined acid and sulfate attack. The precipitation of gypsum at the degradation front can reduce the diffusion of sulfates and acid protons and therefore the rate of degradation. For this reason, the rate of degradation, as well as the quantification of the corrosion by sulfuric acid, is much more influenced by the testing method (that affects the pH and the concentration of sulfate) than for the other acids. Furthermore, in sulfuric acid attack, the decrease in density and the increase in volume of the concrete due to the sulfuric acid reaction with the hydration products can be even larger at higher pH. This means that a severe sulfuric acid attack (very low pH) could produce a smaller weight of loss in a concrete specimen than that produced by a weaker solution (i.e. at pH 4), as observed in previous studies of Attiogbe and Rizkalla [7]. Indeed, at higher sulfuric acid concentrations, gypsum formation dominates and it can fill the pores creating a protective layer; hence, the degradation could be lower than for lower concentrations, at least in the first months of exposure. Contrarily, the increase in weight loss of concrete that is exposed to other acid media is normally proportional to the increase in acidity. For this reason, the loss of weight or the change in volume of concrete subjected to sulfuric acid attack can be much more influenced by the experimental parameters than in the case of other types of acid attack. 

The influence of the cement type and, in particular, the content of C_3_A (that is lower for the CEM II/B-S 52.5 R than for the CEM I 52.5 R, as seen in Table 1), the ratio between the C_3_S/C_2_S, and the use of SCMs can play a relevant role. Nevertheless, one common result emerging from the different test procedures is that the resistance of SCC to severe sulfuric acid attack was moderately improved by the granulometrically optimized slag cement. The high quality of concrete used (high workability, good consolidation, low w/c ratio, low porosity, and low permeability) leads to a minor influence of the type of cement used.

Each test method presented in this study had some practical advantages, as summarized in Table 3. Anyway, the influence of the experimental conditions hinders a direct and quantitative comparison of the results that are expressed by the loss of mass, between the methods that are presented in this paper and those published in the literature. The expression of the loss of mass by kg/m^2^ instead of loss of mass by % related to the initial weight, as often reported in literature, allows for the comparison between concrete samples with different geometry, since the acid attack is related to the surface that is exposed to the solution and not the bulk. 

The brushing or not brushing procedure of the surface is often a matter of discussion in literature; however, many researchers agreed that the brushing is recommended to obtain a reliable comparison between the behavior of different concretes that are subjected to acid attack [18].

The “constant pH method” and “constant sulfate method” use a similar approach, which is based on the immersion of concrete specimens, even if with different geometry, in sulfuric acid solution at pH 2, and a manual brushing of the surface. The direct comparison of the loss of weight obtained after seven months of exposure from the two methods is strongly affected by the differences in the experimental parameters, mainly the frequency of renewal of the solution and the sulfate concentration. In the “constant pH method”, the acid solution is added by a titration mechanism to keep the pH constant, which implies a higher concentration of sulfate when compared to the “constant sulfate method” in which the pH is evaluated manually and adjusted when it goes above pH 4. During the first months, the solution is renewed in both methods weekly and in both cases the brushing is done manually, even if with different brushes and by different operators. This explains the good agreement regarding the loss of mass measured during the first 30 days obtained from the two methods, circa 0.4 kg/m^2^, regardless the sample geometry and the different ratio Va/S. Afterwards, the frequency of the solution renewal in the “constant pH method” is reduced to one time per month; this can easily explain why after seven months the loss of mass is lower than the one that is obtained in the “constant sulfate method”. It can be concluded that the frequency of the solution renewal has the biggest influence on the results, whilst the influence of sample geometry and the ratio between solution volume/specimen surface is less relevant.

Actually, a comparison with the amount of material that is removed by the TAP method can also be made, under the assumption that the swelling of the remaining concrete matrix after brushing is limited. Subsequently, a change in radius of about 1 mm corresponds to a mass loss of 2.338 kg/m² for the SCC-OPC and 2.381 kg/m² for the SCC-Slag (taking the densities in Table 4 into account). The SCC-Slag then shows after 25 weeks (almost six months) of exposure a mass loss of about 2.38 kg/m², which is not all that different from the mass loss after six months for the constant sulfate method (around 2.5 kg/m²). Although in the TAP procedure the samples experience a higher pH value during part of the cycles, the alternated wetting and drying enhances the penetration of the acid into the matrix, which will increase the degradation rate. 

It was observed in both the “constant sulfate method” and in the “TAP method” that the pH of the solution containing the SCC-OPC increased quicker than for SCC-Slag during the exposure to sulfuric acid, due to the higher portlandite content in the concrete with CEM I 52.5R than that with slag addition.

The results of the “TAP method” showed a higher radius reduction of the SCC-OPC due to the acid corrosion as compared to the SCC-Slag. This trend is in agreement with the loss of mass measured by the “constant sulfate method”; the SCC-OPC has a bit higher loss of mass than SCC-Slag. Nevertheless, the observed differences are not substantial and it can be concluded that the high quality of the self-compacting concrete leads to a minor influence of the cement type. In the “constant pH method”, indeed no differences are observed between the two concrete types after seven months of exposure to sulfuric acid solution at pH 2.

## 5. Conclusions

The use of three different acid test methods applied by different research institutes allowed for studying the performance of two SCC made with an ordinary Portland cement CEM I 52.5R or with with granulometrically optimized slag cement CEM II/B-S 52.5R that was subjected to a severe sulfuric acid attack (pH 2). The impact of the experimental conditions were evaluated and discussed. The quantification of the degradation by the loss of mass or volume change of concrete is mainly influenced by the frequency of the acid solution renewal, in which the concretes are immersed and the mechanical brushing process used to remove the degraded layer. The sulfuric acid attack at very low pH is strongly influenced by the precipitation of gypsum crystals, which can induce a long dormant period and act as self-protecting layer that is limiting the diffusion of the acid inside the matrix. For this reason, the two experimental parameters mentioned before were identified as the most relevant for a data comparison between the different methods.

The use of fly-ash might give a positive contribution against acid attack, since it was observed by electron microscopy that the fly ashes withstand the acid attack. The resistance of SCC that was made with OPC and with granulometrically optimized slag cement to sulfuric acid attack at pH 2 was almost comparable. In the case of low w/c ratio and optimized concrete technology, the influence of the cement type becomes less relevant. Nevertheless, a common result that emerges from the different test procedures is that the granulometrically optimized slag cement moderately improves the resistance against sulfuric acid. This observation implies that the use of granulometrically optimized slag cement is recommended to increase the durability of structures exposed to acidic media.

## Figures and Tables

**Table 1:** Chemical and mineralogical analysis of the binders.

Component	CEM II/B-S 52.5 R	CEM I 52.5 R	Fly-Ash	
SiO2	25.7	21.4	52.4	(%)
CaO	57.8	64.7	3.1	(%)
Al2O3	5.9	3.7	24.6	(%)
Fe2O3	1.1	1.3	8.1	(%)
MgO	2.5	0.7	1.9	(%)
K2O	0.5	0.6	3.2	(%)
SO3	2.2	3.4	0.38	(%)
C3S	49.0	70.3		(%)
C2S	5.3	6.5		(%)
C3A	6.9	8.9		(%)
C4AF	1.1	2.4		(%)

**Table 2:** Mix design of the self-compacting concrete (SCC).

Material	Type/Description	SCC-Slag	SCC-OPC	
Cement	CEM II/B-S 52,5R	420		
	CEM I 52.5R		420	kg/m³
SCM	Fly Ash	100	100	kg/m³
Aggregate 1	Sand 0/2	738	732	kg/m³
Aggregate 2	Sand 2/8	302	300	kg/m³
Aggregate 3	Gravel 8/16	637	632	kg/m³
Water	Tap water	165	165	kg/m³
w/c-ratio	--	0.36	0.36	--
Powder content	--	520	520	kg/m³
Superplasticiser	--	0.80	1.0	%bwc
Plasticizer based Slump controller	--	0.18	0.5	%bwc
Surface Improver	--	0.4	0.5	%bwc

**Table 3:** Comparison between the acid test resistance methods.

Test Method	Protocol	Main Features
Constant pH method	Storage of prismatic samples (100 × 150 × 40 mm^3^) in sulfuric acid with continuous pH control and adjustment by an automatic titrator.	Controlled and constant pH. Enhanced sulfate attack. Chance to test different sample geometries. Manual brushing.
Constant sulfate method	Storage of cylindrical concrete (∅ = 75 mm × 20 mm) in separate plastic boxes allows measurement of pH and ions in solution.	Does not need a dedicated set-up. Manual control of the pH. Easy analysis of the leached ions in solution. Manual brushing.
TAP method	Alternating wetting and drying cycles of cylindrical samples (∅ = 230 mm) rotating in a vessel with sulfuric acid	Automatic and reproducible brushing. Simulation of a real condition with wet/dry cycles. Manual control of the pH.

**Table 4:** Compressive strength, dynamic E-modulus, and water adsorption of the concrete mixtures.

Concret	Compressive Strength 28 d [MPa]	Slump Flow (mm)	Static E-Modulus [GPa]	Capillary Water Sorption [M-%]	Density [kg/dm³]	Total Porosity (%-Vol.)
SCC-Slag	93 ± 2	765	41.4 ± 0.9	0.25 ± 0.02	2.381 ± 0.005	4.58
SCC-OPC	87 ± 2	755	36.4 ± 4.4	0.44 ± 0.05	2.338 ± 0.005	5.02
